# An Exploratory Study for Proteomic‐Based Markers of Joint Pain and Chronic Back Pain

**DOI:** 10.1002/ejp.70158

**Published:** 2025-10-31

**Authors:** Tessa Schillemans, Ann‐Sofie Rönnegård, Themistocles L. Assimes, Magnus Peterson, Per Wändell, Lars Lind, Johan Ärnlöv

**Affiliations:** ^1^ Department of Neurobiology, Care Sciences and Society Karolinska Institutet Stockholm Sweden; ^2^ Department of Health and Social Studies Dalarna University Falun Sweden; ^3^ Center for Clinical Research Dalarna Uppsala University, Region Dalarna Falun Sweden; ^4^ Division of Cardiovascular Medicine, Department of Medicine Stanford University School of Medicine Stanford California USA; ^5^ VA Palo Alto Health Care System Palo Alto California USA; ^6^ Cardiovascular Institute, Stanford University School of Medicine Stanford California USA; ^7^ Department of Epidemiology and Population Health Stanford University School of Medicine Stanford California USA; ^8^ Department of Public Health and Caring Sciences Uppsala University Uppsala Sweden; ^9^ Center for Primary Health Care Research Lund University Malmö Sweden; ^10^ Department of Medical Sciences, Clinical Epidemiology Uppsala University Uppsala Sweden

## Abstract

**Background:**

Joint pain and chronic back pain are highly prevalent in the aging population and have a large impact on life quality. As the underlying mechanisms are not fully understood, this exploratory cross‐sectional study aimed to discover proteins and pathways associated with these two pain conditions in Swedish 70‐year‐old men.

**Methods:**

Plasma proteins (*n* = 720) were measured in participants from the Uppsala Longitudinal Study of Adult Men (ULSAM; *n* = 931) using Olink target panels. Participants self‐reported current joint pain or continuous back pain during the past year. We used logistic regression with multiple testing adjustments and RIDGE regression (selecting ~10% highest‐ranking proteins) to identify proteins associated with either joint or chronic back pain, which were then investigated for clusters and pathway enrichments.

**Results:**

Out of 931 subjects with protein data, 131 reported joint pain and 31 reported chronic back pain. We identified 19 (significant after multiple testing adjustment) and 25 (nominally significant) highest‐ranking proteins associated with joint and chronic back pain, respectively. Enriched pathways included immune responses, inflammation, lipid, coagulation and rheumatoid arthritis pathways. Similar pathways were found for both joint and chronic back pain, even though only two proteins were associated with both these pain conditions.

**Conclusions:**

This exploratory proteomics study provides support for systemic inflammation as a common underlying mechanism for joint and chronic back pain. Although similar pathways were found for both pain conditions, the selected proteins differed. Nevertheless, caution is advised due to low sample size and validation in larger studies including both women and men is needed.

**Significance Statement:**

Logistic and RIDGE regression analyses indicated that joint pain and chronic back pain were associated with different proteins, which were enriched for similar inflammatory pathways.

## 1 Introduction

Pain is a complex heterogeneous process that can have many underlying interacting causes, which are currently not well understood. Chronic pain conditions, defined as pain > 3 months, are common (estimated prevalence of 20% (Breivik et al. 2006; Dahlhamer et al. 2018)) and take a large toll on quality of life as well as on socioeconomic costs (Cohen et al. 2021). The most common forms of chronic pain are back pain (> 50%) and joint pain (40%) (Breivik et al. 2006). Joint pain can be related to underlying conditions such as osteoarthritis or rheumatoid arthritis involving mostly inflammatory, but also a range of non‐inflammatory pathways (Havelin and King 2018). Chronic back pain is even more diverse and it can be related to myofascial pain (most common), structural damage, inflammation and neurodegenerative changes (Mosabbir 2022). However, most cases are non‐specific and do not have a clear underlying origin of the pain.

Objective biomarkers indicating underlying biological processes would facilitate diagnosis, management and identification of treatment targets of pain conditions (Gerdle and Ghafouri 2020). However, research using the proteome for pain research is still relatively unexplored and it is not yet known to what degree circulating proteins correlate with patient ‘subjective’ experience of pain sensations and whether different types of pain are associated with different proteomic signatures (Bay‐Jensen et al. 2021; Firdous et al. 2022; Huang and Sowa 2011; Thudium et al. 2019). Current proteomics research has mainly indicated involvement of both metabolic processes and inflammation as underlying mechanisms for pain. However, only a few studies have been performed and most are limited by small sample size, lack of multivariate methods capable of handling collinearity and network analyses investigating clusters rather than individual proteins, and studying only one pain condition which does not allow for protein signature comparisons between conditions (Gerdle and Ghafouri 2020).

Therefore, we aimed to identify proteins associated with joint pain and chronic back pain in a cohort of 70‐year‐old men living in Sweden. We explored both logistic regression with multiple testing adjustment for single protein associations, and RIDGE regression machine learning followed by pathway enrichment analysis to identify the most robust findings and provide complementary perspectives. We also explored whether there was any overlap between proteins and potential underlying networks and pathways related to these two different pain conditions.

## 2 Methods

### 2.1 Study Population

The Uppsala Longitudinal Study of Adult Men (ULSAM) study was initiated in 1970 as a longitudinal cohort study with the aim to investigate risk factors for CVD (Hedstrand 1975). The study comprised all men living in the County of Uppsala born between 1920 and 1924 (aged 50 years) selected from the register of County Council. All men (*n* = 2841) were invited for the investigation and 81.7% (*n* = 2322) participated. Follow‐up was performed at ages 60, 70, 77, 82, 88 and 93. The current study uses data from the 20‐year follow‐up (conducted between 1991 and 1995), including *n* = 1221 men aged 70 years. This follow‐up included a questionnaire (including questions regarding joint and back pain), examination and blood drawing. Of these, 931 participants had data on both pain status and proteomics and thus comprise the present study sample. Informed consent was obtained from the participants and the ULSAM study has been approved by the Ethics Committee of Uppsala University.

### 2.2 Joint Pain and Chronic Back Pain

Joint pain was defined as self‐reported pain in the joints (answering “Yes” to “Are you troubled by pains in the joints?”). Controls were defined as those that did not self‐report pain in the joints (answering “No” or “Do not know” or leaving the question blank).

Chronic back pain was defined as self‐reported chronic (≥ 1 year), continuous lower or upper back pain (answering “Yes” to “Do you ever get pain or other problems in the upper OR lower back?” AND answering any option ≥ 1 year to “How long have you had pain or discomfort in the upper OR lower back?” AND answering “continuous” to “How often do you get pain or discomfort in the upper OR lower back?”). Controls were defined as those that did not self‐report chronic (≥ 1 year), continuous lower or upper back pain (answering “No” or “< 1 year” or “periodically” or leaving blank any of the required above‐mentioned questions).

### 2.3 Proteomics Measurement

Blood samples from the 20‐year follow‐up visit (1991–1995) were drawn into EDTA plasma tubes in the morning after an overnight fast, spun in the centrifuge and within 1 h placed in vials and frozen at −80°C. The proteins were measured in 2020 using antibody‐based Proximity Extension Assay using nine OLINK Target panels (i.e., Cardio‐metabolic, Cardiovascular II, Cardiovascular III, Development, Immune response, Inflammations, Metabolism, Oncology II and Organ damage panels). Each panel includes 92 proteins including established markers as well as exploratory markers (http://www.olink.com/proseekmultiplex/complete‐biomarker‐list/). Two matched antibodies labelled with DNA oligonucleotides simultaneously bind to a target protein in the solution; the close proximity of their DNA oligonucleotides leads to their hybridization and extension by DNA polymerase resulting in a DNA sequence specific for the target protein. This DNA sequence is then amplified and quantified by real‐time quantitative polymerase chain reaction to measure relative changes in protein expression (normalised protein expression (NPX)). Olink uses a quality control system of four internal controls spiked into each sample to monitor the performance of assays and samples during each step. Further details regarding levels of detection, reproducibility and validations are available at: https://www.olink.com/resources‐support/document‐download‐center/. From the total 828 proteins measured, 86 proteins with > 25% of the values below LOD were excluded. Additionally, a small number of proteins were present on more than one panel and these duplications were removed prior to analysis to avoid collinearity issues resulting in a total number of *n* = 720 proteins. Additionally, subjects with missing protein data (due to lack of plasma in the freezer or multiple missing protein data) were excluded resulting in a total number of *n* = 931 subjects. Protein levels were standardised prior to regression analyses and thus results are presented per 1‐SD increase in protein NPX.

### 2.4 Confounders

Analyses were adjusted for age (continuous in years), self‐reported education level at age 50 (4 categories: 7/8 years of education, 12 years of education, college and missing indicator category) and proteomics plate identification number (11 categories) for potential batch differences. In sensitivity analyses, we also adjusted for body mass index (BMI) to investigate the impact of BMI on our results as BMI could be either a confounder or mediator and excluded subjects with C‐reactive protein (CRP) values > 5 mg/L measured in blood to investigate whether outliers with severe inflammation could have influenced our results.

### 2.5 Logistic Regression Models

First, we conducted a logistic regression analysis adjusted for confounders for each individual protein to assess their marginal adjusted associations with joint pain or chronic back pain one by one. Joint pain or chronic back pain was used as the dependent variable whilst each of the 720 proteins was individually used as the independent variable. Resulting *p*‐values were adjusted for multiple testing using the Benjamini‐Hochberg False Discovery Rate (FDR) method. Results are presented in a volcano plot and significant odds ratios (OR), 95% confidence intervals (95% CI), *p*‐values and FDR values are presented in a table.

### 2.6 RIDGE Regression Models

Second, to account for potential collinearity amongst variables and to identify the most predictive features, we performed a simultaneous multivariable penalised analysis (all proteins together) using a RIDGE regression model. RIDGE regression is an extension of a multivariate regression employing regularisation to penalise large coefficients in the model. This leads to a better ability to deal with multicollinearity and helps to avoid overfitting. This allows us to instead of investigating proteins one‐by‐one (as in the logistic regression), model all proteins at once. In contrast to lasso and elastic net, RIDGE does not eliminate variables; thus an arbitrary cut‐off of highly important variables can be chosen for further investigation and pathway analysis. Joint pain or chronic back pain was used as the dependent variable whilst all proteins were entered together as independent variables. The models were adjusted for age, education level and protein plate number by including them in the independent variables using a penalty factor to ensure they remain in the model and using one‐hot encoding on the categorical variables (education and protein plate number; R package caret). Weights were used to adjust for dataset imbalance. Alpha was set as 0 and the RIDGE regularisation parameter lambda was selected over a total of 100 folds in cross‐validation (R package: glmnet). Features were selected from the weighted RIDGE regression model using the lambda value that gave the minimum cross‐validation error (lambda.min). Features were selected using an arbitrary coefficient cut‐off selecting approximately ~10% of proteins.

Model performance was evaluated using:

1. Regularisation and cross‐validation plots.
2. Predicting ROC curves and AUC.
3. Investigating confusion matrix and distributions of predicted probabilities to actual values to see how well the model is able to predict joint pain.
4. Splitting into train and test data (80/20%) and evaluating performance (1–3) on test data.

Subsequently, we investigated protein enrichment pathways using the R package “gprofiler2” (Kolberg et al. 2020; Raudvere et al. 2019) using KEGG and *homo sapiens* databases (accessed on 28‐08‐2025). Default methods were used for multiple testing adjustment (Bonferroni) and significance (0.05 cutoff). Adjusted *p*‐value was plotted on the *x*‐axis whilst precision (proteins in query in the specific pathway/total number of proteins in a specific pathway) was indicated by point size and recall (proteins in query in the specific pathway/total number of proteins in query) by color.

### 2.7 Intersection, Visualisation and Protein Investigation

Third, we combined results from both logistic and RIDGE regression approaches by further investigating the proteins that were selected in both methods. Combining both these approaches (focusing on single proteins versus proteins modelled jointly) allowed for a more layered evaluation, providing complementary perspectives and enhancing the robustness and interpretability of the results. Intersection proteins were visualised for their intercorrelations in a heatmap (R package: corrplot). We furthermore employed a STRING search (accessed on 09‐05‐2025) including all proteins from both pain conditions to investigate potential protein enrichment pathways and clusters. The following settings were used for the network: 1. Full STRING network of both functional and physical protein associations; 2. Active interaction sources including textmining, experiments, databases, co‐expression, neighbourhood, gene fusion and co‐occurrence; 3. Minimum required interaction score = 0.4 for medium confidence; 4. No interactors.

### 2.8 Statistical Program

R studio version 4.4.2 was used for all analyses.

## 3 Results

### 3.1 Population Descriptive

We observed 131 subjects that reported joint pain versus 800 remaining subjects that did not report joint pain. Additionally, we observed 31 that reported chronic back pain versus 900 remaining subjects that did not. Overall, we did not observe large differences in age or in education level, although subjects with joint pain had a lower percentage in the higher education levels compared to the controls without joint pain (Table 1). However, subjects with either pain condition had higher levels of BMI and CRP (Table 1).

**TABLE 1:** Description of ULSAM subjects with available proteomics data (*n* = 931) comparing those with and without joint pain or chronic back pain.

	No joint pain (*N* = 800)	Joint pain (*N* = 131)	No chronic back pain (*N* = 900)	Chronic back pain (*N* = 31)	Overall (*N* = 931)
Age					
Mean (SD)	71.0 (0.651)	71.0 (0.618)	71.0 (0.650)	71.0 (0.515)	71.0 (0.646)
Education, *n* (%)					
7/8 years	521 (65.1%)	99 (75.6%)	599 (66.6%)	21 (67.7%)	620 (66.6%)
12 years	43 (5.4%)	4 (3.1%)	44 (4.9%)	3 (9.7%)	47 (5.0%)
College	87 (10.9%)	8 (6.1%)	91 (10.1%)	4 (12.9%)	95 (10.2%)
Missing data	149 (18.6%)	20 (15.3%)	166 (18.4%)	3 (9.7%)	169 (18.2%)
Physical activity, *n* (%)					
1	21 (2.6%)	11 (8.4%)	28 (3.1%)	4 (12.9%)	32 (3.4%)
2	246 (30.8%)	37 (28.2%)	274 (30.4%)	9 (29.0%)	283 (30.4%)
3	390 (48.8%)	68 (51.9%)	444 (49.3%)	14 (45.2%)	458 (49.2%)
4	47 (5.9%)	7 (5.3%)	52 (5.8%)	2 (6.5%)	54 (5.8%)
Missing, *n* (%)	96 (12.0%)	8 (6.1%)	102 (11.3%)	2 (6.5%)	104 (11.2%)
BMI					
Mean (SD)	26.1 (3.25)	27.7 (4.05)	26.3 (3.36)	27.1 (4.85)	26.3 (3.42)
Missing, *n* (%)	2 (0.3%)	1 (0.8%)	3 (0.3%)	0 (0%)	3 (0.3%)
CRP					
Mean (SD)	3.23 (4.71)	4.74 (5.66)	3.38 (4.82)	5.27 (6.14)	3.44 (4.88)
Missing, *n* (%)	26 (3.3%)	3 (2.3%)	29 (3.2%)	0 (0%)	29 (3.1%)

*Note:* Continuous variables are presented as mean (SD) and categorical as *n* (%). There are 13 people from the chronic back pain group (42%) also appearing in the joint pain group.

Abbreviations: BMI, body mass index; CRP, C‐reactive protein.

### 3.2 Logistic Regression Results

**FIGURE 1:**
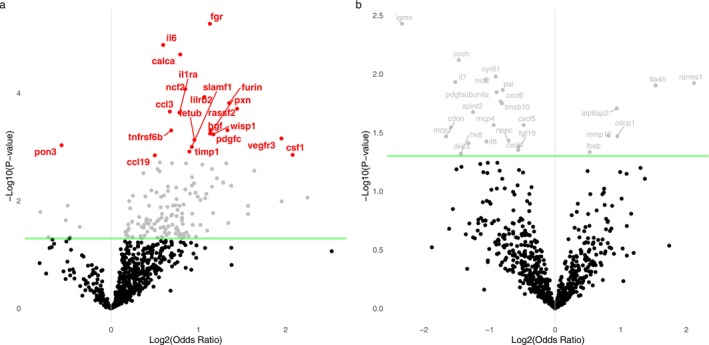
Volcano plot presenting the results from logistic regression with 720 unscaled proteins for (a) joint pain and (b) chronic back pain. Green horizontal line and grey dots and labels present nominal significance (*p*‐value < 0.05) whilst red coloured points and labels present FDR significant findings (FDR *p*‐value < 0.05).

Logistic regression including 931 subjects and 720 proteins resulted in 21 proteins significantly associated with joint pain after adjustment for age, education and protein plate number (FDR *p*‐value < 0.05), of which only one was associated inversely (Figure 1a and Table 2). Additional adjustments for BMI as well as exclusion of subjects with CRP ≥ 5 mg/L had minor to moderate impact on the ORs, but only IL6RA and PON3 were no longer significantly associated after BMI adjustment and CRP exclusion (only PON3) (Table 2). We did not observe any proteins significantly associated with chronic back pain in the main model after FDR adjustment, although there were 26 proteins nominally associated (Figure 1b).

**TABLE 2:** Associations from logistic regression between joint pain and proteins.

Uniprot protein ID	Protein Name	OR (95% CI)	*p*	FDR *p*	Adjusted for BMI^a^ OR (95% CI)	Excluded CRP ≥ 5^b^ OR (95% CI)
P01033	timp1	1.32 (1.11–1.56)	0.0012	0.0462	1.29 (1.08–1.53)	1.31 (1.07–1.61)
Q9UGM5	fetub	1.37 (1.14–1.65)	7e−04	0.0333	1.30 (1.08–1.57)	1.35 (1.08–1.68)
Q8N423	lilrb2	1.43 (1.19–1.72)	1e−04	0.0171	1.38 (1.15–1.66)	1.51 (1.21–1.89)
P18510	il1ra	1.41 (1.17–1.69)	2e−04	0.0185	1.19 (0.98–1.46)	1.37 (1.10–1.71)
P05231	il6	1.45 (1.23–1.71)	0	0.0046	1.37 (1.15–1.62)	1.29 (1.06–1.58)
P10147	ccl3	1.37 (1.16–1.62)	2e−04	0.0185	1.35 (1.14–1.61)	1.29 (1.05–1.57)
Q15166	pon3	0.74 (0.62–0.89)	9e−04	0.0393	0.84 (0.69–1.02)	0.76 (0.61–0.94)
Q13291	slamf1	1.35 (1.13–1.61)	0.001	0.0399	1.28 (1.06–1.53)	1.28 (1.03–1.58)
Q99731	ccl19	1.33 (1.11–1.58)	0.0014	0.0491	1.28 (1.06–1.53)	1.31 (1.06–1.61)
P14210	hgf	1.39 (1.16–1.68)	5e−04	0.0296	1.28 (1.06–1.56)	1.42 (1.13–1.78)
P09603	csf1	1.37 (1.13–1.66)	0.0014	0.0491	1.30 (1.07–1.59)	1.35 (1.08–1.71)
O95407	tnfrsf6b	1.36 (1.14–1.63)	5e−04	0.0296	1.34 (1.12–1.60)	1.32 (1.07–1.63)
P09958	furin	1.46 (1.20–1.78)	2e−04	0.0184	1.27 (1.03–1.56)	1.43 (1.13–1.81)
O95388	wisp1	1.37 (1.14–1.63)	6e−04	0.03	1.30 (1.08–1.57)	1.49 (1.20–1.84)
P35916	vegfr3	1.45 (1.18–1.80)	7e−04	0.0333	1.36 (1.11–1.70)	1.49 (1.16–1.95)
P49023	pxn	1.39 (1.15–1.69)	6e−04	0.03	1.34 (1.11–1.63)	1.33 (1.06–1.67)
Q9NRA1	pdgfc	1.42 (1.17–1.74)	5e−04	0.0296	1.36 (1.12–1.66)	1.37 (1.07–1.75)
Q9Y6F1	rassf2	1.37 (1.16–1.61)	2e−04	0.0185	1.37 (1.16–1.62)	1.39 (1.14–1.68)
P01258	calca	1.47 (1.23–1.77)	0	0.0046	1.40 (1.17–1.69)	1.42 (1.14–1.76)
P19878	ncf2	1.42 (1.19–1.70)	1e−04	0.0152	1.41 (1.18–1.69)	1.36 (1.10–1.68)
P06241	fgr	1.49 (1.25–1.77)	0	0.0037	1.49 (1.25–1.77)	1.43 (1.17–1.75)

*Note:* Results are presented only for significant proteins (FDR < 0.05) per 1‐SD increase in NPX protein level.

^a^ Main model additionally adjusted for BMI (continuous variable); missing data for 1 joint pain and 2 controls were removed.

^b^ Main model including only subjects with CRP values < 5 (*n* = 722: *n* = 633 controls and *n* = 89 joint pain). Abbreviations: BMI = Body Mass Index; CRP = C‐Reactive Protein; FDR = False Discovery Rate; OR = Odds Ratio; 95% CI = 95% Confidence Interval.

### 3.3 RIDGE Regression Results: Joint Pain

Weighted RIDGE regression including 931 subjects and 720 proteins penalized for age, education and protein plate number resulted in a final selection of 87 proteins associated with joint pain (lambda.min = 5.47 and coefficient cut‐off = 0.005). Model performance and cross‐validation metrics are reported in Figure S2. Out of these 87 proteins, 19 matched with significant findings from the logistic regression (Figure S3).

**FIGURE 2:**
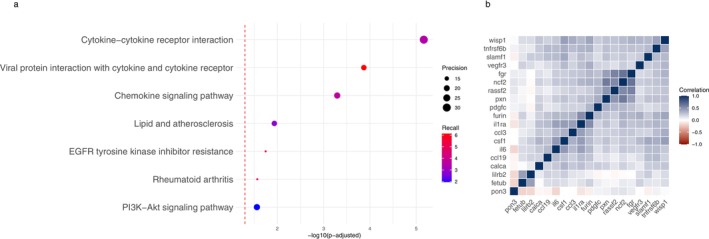
Proteins and pathways associated with joint pain. (a) Pathway enrichment analysis based on all upregulated proteins selected in RIDGE regression (coefficient > 0.005; Bonferroni < 0.05). (b) Heatmap of correlations between proteins selected in both logistic and RIDGE regression.

Pathway analysis on the 58 upregulated proteins indicated 7 significant enriched pathways including inflammatory, lipid and rheumatoid arthritis pathways (Figure 2a), but pathway analysis on the 29 downregulated proteins indicated no significant enriched pathways. Correlation analysis of the 19 proteins selected in RIDGE and significantly associated with joint pain in logistic regression is visualised in Figure 2b.

### 3.4 RIDGE Regression Results: Chronic Back Pain

**FIGURE 3:**
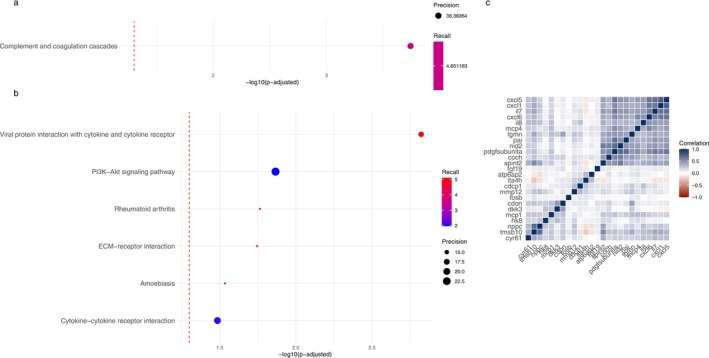
Proteins and pathways associated with chronic back pain. (a) Pathway enrichment analysis based on all upregulated proteins selected in RIDGE regression (coefficient > 0.0005; Bonferroni < 0.05). (b) Pathway enrichment analysis based on all downregulated proteins selected in RIDGE regression (coefficient < −0.0005; Bonferroni < 0.05). (c) Heatmap of correlations between proteins selected in both logistic and RIDGE regression.

Using weighted RIDGE regression including 931 subjects and 720 proteins penalised for age, education and protein plate number, we selected 78 proteins (lambda.min = 122.7 and coefficient = 0.0005). Model performance and cross‐validation metrics are reported in Figure S4. Out of 26 nominally significant proteins in the logistic regression analysis, 25 proteins were also manually selected in the weighted RIDGE regression (Figure S5). Pathway analysis indicated complement and coagulation cascades and inflammatory pathways (Figure 3a,b). Correlations between the 25 proteins are visualised in Figure 3c.

**FIGURE 4:**
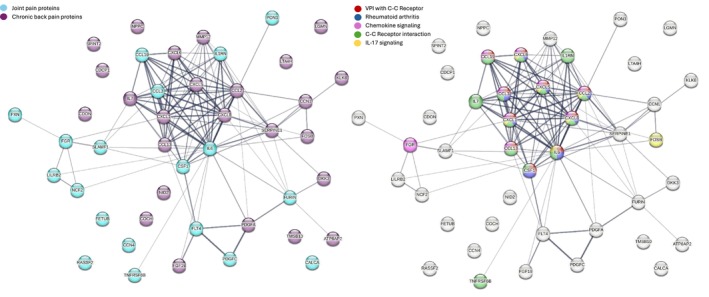
Proteins and pathways associated with joint pain (*n* = 19) and chronic back pain (*n* = 25) together in a STRING analysis (PPI enrichment *p* < 1.0e−16). Proteins are coloured by (a) joint pain or chronic back pain findings and (b) pathway (5 most significant KEGG pathways, FDR < 0.05). Abbreviations: C‐C, cytokine‐cytokine; VPI, Viral protein interaction.

Interestingly, none of the intersection proteins of joint pain overlapped with those found for chronic back pain and only 2 proteins from the larger selection from RIDGE regression overlapped (FETUB and SERPINA12) (selected proteins are listed in Figure S6). Yet, several pathways were found for both joint pain and chronic back pain proteins (Figures 2d and 3d,e) and investigating all proteins selected from RIDGE from both joint pain and chronic back pain indicated significant protein–protein interactions (PPI *p*‐value < 1.0e−16) and several enriched KEGG pathways (Figure 4).

## 4 Discussion

### 4.1 Summary

The present exploratory study aimed to identify proteins underlying joint pain and chronic back pain to increase our understanding of molecular mechanisms underlying these two pain conditions. By using two different methodological approaches, (1) traditional statistical analysis investigating proteins one by one and (2) machine learning analysis investigating all proteins together followed by pathway enrichment analysis, we aimed to provide complementary perspectives. We found several proteins related to joint pain (*n* = 19 selected in both logistic (FDR < 0.05) and RIDGE (coefficient > |0.005|) regression models), whilst chronic back pain was more challenging to model (*n* = 25 nominal significance [*p* < 0.05] in logistic and RIDGE [coefficient > |0.0005|] regression models) likely due to large class imbalances and too few subjects in the chronic back pain group. None of the proteins FDR significant for joint pain overlapped with the nominally significant proteins for chronic back pain and only 2 proteins from the highest ranking proteins in the RIDGE models were found for both joint pain and chronic back pain. Yet, the selected proteins could be mapped to the same pathways mainly related to inflammation for both pain conditions.

### 4.2 Pain Proteins and Mechanisms

There are a few review studies on proteomics, biomarkers or signalling pathways for pain conditions in humans, including chronic pain (Gerdle and Ghafouri 2020), low back pain (Lim et al. 2020), osteoarthritis (Fu et al. 2022; Welhaven et al. 2025), rheumatoid arthritis (Ding et al. 2023) and fibromyalgia (Gkouvi et al. 2024). Most of these conditions have previously been shown to be associated with similar pathways including inflammation, oxidative stress, lipid metabolism, extracellular matrix interaction and tissue remodelling. However, the associations with individual proteins differed between the conditions (Gerdle and Ghafouri 2020). Interestingly, our findings indicated no overlap between FDR significant proteins for joint pain with the nominal significant proteins for chronic back pain but highlighted many of the same pathways. This may indicate involvement of unique proteins between these pain conditions, which ultimately affect similar pathways. However, caution is required since our sample size was limited and no FDR significant chronic back pain proteins were found. Additionally, individual proteins may be harder to replicate whilst processes may be more robust across different studies (Gerdle and Ghafouri 2020), which could also explain findings of shared pathways, but distinct individual proteins between pain conditions.

Our finding for rheumatoid arthritis pathways, immune response and inflammatory pathways (i.e., rheumatoid arthritis, viral protein interaction and cytokine‐cytokine receptor, cytokine‐cytokine receptor interaction, chemokine signalling pathway) is in line with current literature indicating inflammatory pathways in relation to joint pain and arthritis (Lin et al. 2018). In addition to this, we found protein enrichment in the PI3K‐Akt signalling pathway and the EGFR tyrosine kinase inhibitor resistance pathway. There is interplay between these pathways and they are involved in regulating proliferation, metabolism, angiogenesis, tissue remodelling and cell survival. Previous research has shown their correlation with the occurrence and development of rheumatoid arthritis and joint injury via chondrocyte hypertrophy, stimulating inflammation and promoting angiogenesis (Ding et al. 2023; Ferrao Blanco et al. 2021; Swanson et al. 2012; Wei et al. 2021).

For joint pain, we also found proteins enriched in the lipid and atherosclerosis pathway. The connection between lipid homeostasis and inflammation has become much investigated during the past decades. There are indications for a bidirectional feedback loop, with inflammation disrupting lipid metabolism and dysregulated lipid metabolism exacerbating inflammation (Sukhorukov and Orekhov 2024). Furthermore, dyslipidemia is hypothesized to damage the joints via decreased nutritional quality to the joint tissues (Blanco 2018) and the lipid and atherosclerosis pathway has been found before in knee osteoarthritis synovial tissue gene enrichment analysis (Xu et al. 2024). Notably, due to the observed association between joint pain and systemic inflammation, joint pain could be a risk factor for cardiovascular disease. In fact, increased cardiovascular disease rates ranging between 1.5 and 2.0‐fold for rheumatoid arthritis and osteoarthritis have been shown before (Solomon et al. 2006; Wang et al. 2016), but for many other pain conditions it is still relatively unknown.

For chronic back pain, the complement and coagulation cascades pathway was found as an enriched pathway amongst the upregulated proteins. This pathway is a key system for healing after injury and it has crosstalk with inflammation, neuronal development and synaptic plasticity. Aberrant activity may lead to neurological deficits and chronic pain and this pathway has been found before in osteoarthritis (Fu et al. 2022; Lin et al. 2018) and fibromyalgia patients (Han et al. 2020; Wåhlén et al. 2020). Inflammation and neuroinflammation are considered one of the major pathways in myofascial pain, the most common type of back pain (Lam et al. 2024). Another enriched pathway that points towards immune system involvement was amoebiasis, an infectious disease, which has been found prior in relation to osteoarthritis (Fu et al. 2022). Lastly, we observed enrichments in the ECM receptor interaction pathway. Extracellular matrix homeostasis is important for intervertebral disc function and dysregulation is considered a hallmark of degeneration (Fu et al. 2022).

Additionally, we will briefly highlight several proteins individually that were selected in our models and have been found in literature before, making them interesting targets for future studies. IL‐6, CSF‐1 have been highlighted in a previous proteomics study on fibromyalgia (Gkouvi et al. 2024). Additionally, many of the other proteins have been indicated in arthritis or pain conditions such as VEGFR3, FGR, TNFRSF6B, FGF19, DKK3 and proteins belonging to CCL, MMP and CXCL families. Most of these are involved in pathways related to inflammation and tissue remodelling (complement and coagulation, lipid metabolism, angiogenesis and oxidative stress).

### 4.3 Strengths and Limitations

This study has several important strengths including the investigation of different types of pain (joint pain and chronic back pain), assessment of a large number of proteins, use of both marginally adjusted models (proteins one‐by‐one) and simultaneous multivariable penalised methods (all proteins together) and investigating potential protein networks and biological pathways. Compared to previous studies reporting associations between pain and proteomics, our relatively large sample size provided better statistical power and robustness.

Nevertheless, this study also suffers from limitations. The main limitation is the imbalanced datasets with a small sample size for the exposed groups. We only identified 31 participants with chronic low back pain, which is lower than estimated in the general population. As our data is self‐reported and we set rather strict criteria, it is possible that there is some underreporting leading to misclassification for controls suffering from chronic back pain. Additionally, participants in this study may be healthier compared to the general population, particularly since our data represents the re‐examination of the original cohort; this may limit the generalizability of the findings. This small sample size may have contributed to our overall null findings in the logistic regression and weak performance of the RIDGE regression models for chronic back pain, even after attempting to balance the dataset using weighting. However, it is also possible that chronic back pain is more difficult to predict using proteomic signatures, as it may have a more diverse aetiology. In line with this, we only have self‐reported data on joint pain and chronic back pain from a questionnaire that was not specifically designed to optimally reflect different aspects of pain severity, duration and widespreadness. The proteomics measurement was performed approximately 25 years after blood sampling; thus there may have been some protein degradation. As all samples share similar storage time, this is unlikely to have caused a differential misclassification or systemic bias between the pain group and reference group, but it is possible that fast degrading proteins may have been missed. Additionally, this is a cross‐sectional study and causality can therefore not be established. Although we currently lack validation data, we plan to further investigate chronic pain in relation to omics data in future studies. Lastly, as ULSAM is a male‐only cohort with similar ages, we were unable to investigate women or other age groups whilst women are overrepresented among chronic pain patients. Thus, additional studies are needed to assess generalizability.

### 4.4 Clinical Implications

Although our findings do not have immediate clinical implications, additional large‐scale studies could identify specific blood proteomic signatures for different pain conditions. This could lead to a deeper understanding of the molecular pathways underlying pain with different origins and thus uncover potential targets for novel therapeutic interventions.

## 5 Conclusion

This exploratory study on proteomics and joint pain and chronic back pain using both traditional logistic regression, investigating single proteins, and machine learning RIDGE regression, investigating proteins jointly, followed by pathway enrichment analysis confirmed and extended our understanding of the pain‐proteomic interplay. The findings furthermore highlight the need for larger sample size studies on pain and omics data.

## Author Contributions

**J.Ä., L.L.** and **T.L.A.** were involved in study design and proteomics measurement. **T.S.** performed the analysis and drafted the manuscript. **A.‐S.R., P.W.** and **M.P.** edited the manuscript. All authors reviewed the results and approved the final manuscript.

## Conflicts of Interest

The authors declare no conflicts of interest. Johan Ärnlöv has received lecture fees from AstraZeneca and Boehringer Ingelheim and served on advisory boards for AstraZeneca, Boehringer Ingelheim and Astella, all unrelated to the present project.

## Supporting information


**Figure S1:** Overview of number of subjects and data analysis.
**Figure S2:** RIDGE model visualisation and performance metrics for joint pain and proteomics. (a) Regularisation plot, (b) Cross‐validation curve, (c) ROC curve for training data (AUC = 0.73; prediction‐recall curve AUC = 0.64), (d) Distribution of predicted probabilities for training data, (e) ROC curve for test data (AUC test = 0.60; prediction‐recall curve AUC test = 0.67) and (f) Distribution of predicted probabilities for test data.
**Figure S3:**. Boxplots for 19 proteins associated with joint pain in both logistic regression (multiple testing adjusted significant) and in RIDGE regression (coefficient cut‐off = |0.005|) with raw data points superimposed.
**Figure S4:** RIDGE model visualisation and performance metrics for chronic back pain and proteomics. (a) Regularisation plot, (b) Cross‐validation curve, (c) ROC curve for training data (AUC = 0.71; prediction‐recall curve AUC = 0.75), (d) Distribution of predicted probabilities for training data, (e) ROC curve for test data (AUC test = 0.55; prediction‐recall curve AUC test = 0.72) and (f) Distribution of predicted probabilities for test data.
**Figure S5:** Boxplots for 25 proteins associated with chronic back pain in both logistic regression (nominally significant) and in RIDGE regression (coefficient cut‐off = |0.0005|) with raw data points superimposed.
**Figure S6:** Protein features selected in RIDGE regression with (a) joint pain (coefficient cut‐off = |0.005|) and (b) chronic back pain (coefficient cut‐off = |0.0005|).

## References

[ejp70158-bib-0001] Bay‐Jensen, A. C. , A. S. Siebuhr , D. Damgaard , et al. 2021. “Objective and Noninvasive Biochemical Markers in Rheumatoid Arthritis: Where Are We and Where Are We Going?” Expert Review of Proteomics 18, no. 3: 159–175. 10.1080/14789450.2021.1908892. 33783300

[ejp70158-bib-0002] Blanco, F. J. 2018. “Osteoarthritis and Atherosclerosis in Joint Disease.” Reumatología Clínica (English Edition) 14, no. 5: 251–253. 10.1016/j.reumae.2018.08.001. 30205874

[ejp70158-bib-0003] Breivik, H. , B. Collett , V. Ventafridda , R. Cohen , and D. Gallacher . 2006. “Survey of Chronic Pain in Europe: Prevalence, Impact on Daily Life, and Treatment.” European Journal of Pain 10, no. 4: 287–333. 10.1016/j.ejpain.2005.06.009. 16095934

[ejp70158-bib-0004] Cohen, S. P. , L. Vase , and W. M. Hooten . 2021. “Chronic Pain: An Update on Burden, Best Practices, and New Advances.” Lancet 397, no. 10289: 2082–2097. 10.1016/s0140-6736(21)00393-7. 34062143

[ejp70158-bib-0005] Dahlhamer, J. , J. Lucas , C. Zelaya , et al. 2018. “Prevalence of Chronic Pain and High‐Impact Chronic Pain Among Adults—United States, 2016.” MMWR. Morbidity and Mortality Weekly Report 67, no. 36: 1001–1006. 10.15585/mmwr.mm6736a2. 30212442 PMC6146950

[ejp70158-bib-0006] Ding, Q. , W. Hu , R. Wang , et al. 2023. “Signaling Pathways in Rheumatoid Arthritis: Implications for Targeted Therapy.” Signal Transduction and Targeted Therapy 8, no. 1: 68. 10.1038/s41392-023-01331-9. 36797236 PMC9935929

[ejp70158-bib-0007] Ferrao Blanco, M. N. , H. Domenech Garcia , L. Legeai‐Mallet , and G. J. V. M. van Osch . 2021. “Tyrosine Kinases Regulate Chondrocyte Hypertrophy: Promising Drug Targets for Osteoarthritis.” Osteoarthritis and Cartilage 29, no. 10: 1389–1398. 10.1016/j.joca.2021.07.003. 34284112

[ejp70158-bib-0008] Firdous, A. , V. Gopalakrishnan , N. Vo , and G. Sowa . 2022. “Challenges and Opportunities for Omics‐Based Precision Medicine in Chronic Low Back Pain.” European Spine Journal. 10.1007/s00586-022-07457-8. 36565345

[ejp70158-bib-0009] Fu, G. , X. Chen , L. Li , et al. 2022. “Function and Pathway Analysis of the Differential Expression Proteins in Osteoarthritis Based on Proteomics Technology.” Osteoarthritis and Cartilage 30: S312–S315. 10.1016/j.joca.2022.02.417.

[ejp70158-bib-0010] Gerdle, B. , and B. Ghafouri . 2020. “Proteomic Studies of Common Chronic Pain Conditions—A Systematic Review and Associated Network Analyses.” Expert Review of Proteomics 17, no. 6: 483–505. 10.1080/14789450.2020.1797499. 32684010

[ejp70158-bib-0011] Gkouvi, A. , S. G. Tsiogkas , D. P. Bogdanos , H. Gika , D. G. Goulis , and M. G. Grammatikopoulou . 2024. “Proteomics in Patients With Fibromyalgia Syndrome: A Systematic Review of Observational Studies.” Current Pain and Headache Reports 28, no. 7: 565–586. 10.1007/s11916-024-01244-4. 38652420 PMC11271354

[ejp70158-bib-0012] Han, C.‐L. , Y.‐C. Sheng , S.‐Y. Wang , Y.‐H. Chen , and J.‐H. Kang . 2020. “Serum Proteome Profiles Revealed Dysregulated Proteins and Mechanisms Associated With Fibromyalgia Syndrome in Women.” Scientific Reports 10, no. 1: 12347. 10.1038/s41598-020-69271-w. 32704114 PMC7378543

[ejp70158-bib-0013] Havelin, J. , and T. King . 2018. “Mechanisms Underlying Bone and Joint Pain.” Current Osteoporosis Reports 16, no. 6: 763–771. 10.1007/s11914-018-0493-1. 30370434 PMC6554716

[ejp70158-bib-0014] Hedstrand, H. 1975. “A Study of Middle‐Aged Men With Particular Reference to Risk Factors for Cardiovascular Disease.” Upsala Journal of Medical Sciences. Supplement 19: 1–61. 1216390

[ejp70158-bib-0015] Huang, W. , and G. Sowa . 2011. “Biomarker Development for Musculoskeletal Diseases.” PM&R 3, no. 6S: S39–S44. 10.1016/j.pmrj.2011.04.023. 21703579

[ejp70158-bib-0017] Kolberg, L. , U. Raudvere , I. Kuzmin , J. Vilo , and H. Peterson . 2020. “gprofiler2—An R Package for Gene List Functional Enrichment Analysis and Namespace Conversion Toolset g:Profiler [Version 2; Peer Review: 2 Approved].” F1000Research 9: 709. 10.12688/f1000research.24956.2. PMC7859841 33564394

[ejp70158-bib-0018] Lam, C. , V. T. Francio , K. Gustafson , M. Carroll , A. York , and A. L. Chadwick . 2024. “Myofascial Pain—A Major Player in Musculoskeletal Pain.” Best Practice & Research Clinical Rheumatology 38, no. 1: 101944. 10.1016/j.berh.2024.101944. 38644073

[ejp70158-bib-0019] Lim, Y. Z. , Y. Wang , F. M. Cicuttini , et al. 2020. “Association Between Inflammatory Biomarkers and Nonspecific Low Back Pain: A Systematic Review.” Clinical Journal of Pain 36, no. 5: 379–389. 10.1097/ajp.0000000000000810. 31990692

[ejp70158-bib-0020] Lin, J. , G. Wu , Z. Zhao , et al. 2018. “Bioinformatics Analysis to Identify Key Genes and Pathways Influencing Synovial Inflammation in Osteoarthritis.” Molecular Medicine Reports 18, no. 6: 5594–5602. 10.3892/mmr.2018.9575. 30365099 PMC6236257

[ejp70158-bib-0021] Mosabbir, A. 2022. “Mechanisms Behind the Development of Chronic Low Back Pain and Its Neurodegenerative Features.” Life (Basel) 13, no. 1: 84. 10.3390/life13010084. 36676033 PMC9862392

[ejp70158-bib-0022] Raudvere, U. , L. Kolberg , I. Kuzmin , et al. 2019. “G:Profiler: A Web Server for Functional Enrichment Analysis and Conversions of Gene Lists (2019 Update).” Nucleic Acids Research 47, no. W1: W191–W198. 10.1093/nar/gkz369. 31066453 PMC6602461

[ejp70158-bib-0023] Solomon, D. H. , N. J. Goodson , J. N. Katz , et al. 2006. “Patterns of Cardiovascular Risk in Rheumatoid Arthritis.” Annals of the Rheumatic Diseases 65, no. 12: 1608–1612. 10.1136/ard.2005.050377. 16793844 PMC1798453

[ejp70158-bib-0024] Sukhorukov, V. N. , and A. N. Orekhov . 2024. “Molecular Aspects of Inflammation and Lipid Metabolism in Health and Disease: The Role of the Mitochondria.” International Journal of Molecular Sciences 25, no. 12: 6299. https://www.mdpi.com/1422‐0067/25/12/6299. 38928004 10.3390/ijms25126299 PMC11204260

[ejp70158-bib-0026] Swanson, C. D. , E. H. Akama‐Garren , E. A. Stein , et al. 2012. “Inhibition of Epidermal Growth Factor Receptor Tyrosine Kinase Ameliorates Collagen‐Induced Arthritis.” Journal of Immunology 188, no. 7: 3513–3521. 10.4049/jimmunol.1102693. PMC3311775 22393153

[ejp70158-bib-0027] Thudium, C. S. , H. Löfvall , M. A. Karsdal , A. C. Bay‐Jensen , and A. R. Bihlet . 2019. “Protein Biomarkers Associated With Pain Mechanisms in Osteoarthritis.” Journal of Proteomics 190: 55–66. 10.1016/j.jprot.2018.04.030. 29704569

[ejp70158-bib-0028] Wåhlén, K. , M. Ernberg , E. Kosek , K. Mannerkorpi , B. Gerdle , and B. Ghafouri . 2020. “Significant Correlation Between Plasma Proteome Profile and Pain Intensity, Sensitivity, and Psychological Distress in Women With Fibromyalgia.” Scientific Reports 10, no. 1: 12508. 10.1038/s41598-020-69422-z. 32719459 PMC7385654

[ejp70158-bib-0029] Wang, H. , J. Bai , B. He , X. Hu , and D. Liu . 2016. “Osteoarthritis and the Risk of Cardiovascular Disease: A Meta‐Analysis of Observational Studies.” Scientific Reports 6: 39672. 10.1038/srep39672. 28004796 PMC5177921

[ejp70158-bib-0030] Wei, Y. , L. Luo , T. Gui , et al. 2021. “Targeting Cartilage EGFR Pathway for Osteoarthritis Treatment.” Science Translational Medicine 13, no. 576: eabb3946. 10.1126/scitranslmed.abb3946. 33441426 PMC8027922

[ejp70158-bib-0031] Welhaven, H. D. , A. H. Welfley , and R. K. June . 2025. “Osteoarthritis Year in Review 2024: Molecular Biomarkers of Osteoarthritis.” Osteoarthritis and Cartilage 33, no. 1: 67–87. 10.1016/j.joca.2024.10.003. 39427749 PMC11663115

[ejp70158-bib-0032] Xu, L. , J. Ma , C. Zhou , et al. 2024. “Identification of Key Hub Genes in Knee Osteoarthritis Through Integrated Bioinformatics Analysis.” Scientific Reports 14, no. 1: 22437. 10.1038/s41598-024-73188-z. 39341952 PMC11439059

