# Supplementary material for: An Exploratory Study for Proteomic‐Based Markers of Joint Pain and Chronic Back Pain
Source: Eur J Pain. 2025 Oct 31;29(10):e70158. doi: 10.1002/ejp.70158 (PMC12576870; doi:10.1002/ejp.70158)
Supplement: Supplementary file 1 — Figure S1: Overview of number of subjects and data analysis. Figure S2: RIDGE model visualisation and performance metrics for joint pain and proteomics. (a) Regularisation plot, (b) Cross‐validation curve, (c) ROC curve for training data (AUC = 0.73; prediction‐recall curve AUC = 0.64), (d) Distribution of predicted probabilities for training data, (e) ROC curve for test data (AUC test = 0.60; prediction‐recall curve AUC test = 0.67) and (f) Distribution of predicted probabilities for test data. Figure S3:. Boxplots for 19 proteins associated with joint pain in both logistic regression (multiple testing adjusted significant) and in RIDGE regression (coefficient cut‐off = |0.005|) with raw data points superimposed. Figure S4: RIDGE model visualisation and performance metrics for chronic back pain and proteomics. (a) Regularisation plot, (b) Cross‐validation curve, (c) ROC curve for training data (AUC = 0.71; prediction‐recall curve AUC = 0.75), (d) Distribution of predicted probabilities for training data, (e) ROC curve for test data (AUC test = 0.55; prediction‐recall curve AUC test = 0.72) and (f) Distribution of predicted probabilities for test data. Figure S5: Boxplots for 25 proteins associated with chronic back pain in both logistic regression (nominally significant) and in RIDGE regression (coefficient cut‐off = |0.0005|) with raw data points superimposed. Figure S6: Protein features selected in RIDGE regression with (a) joint pain (coefficient cut‐off = |0.005|) and (b) chronic back pain (coefficient cut‐off = |0.0005|). [file EJP-29-0-s001.docx]

# Supplemental information

Belonging to the manuscript: “An exploratory study for proteomic based markers of joint pain and chronic back pain” by Tessa Schillemans, Ann-Sofie Rönnegård, Themistocles L. Assimes, Magnus Peterson, Per Wändell, Lars Lind and Johan Ärnlöv.


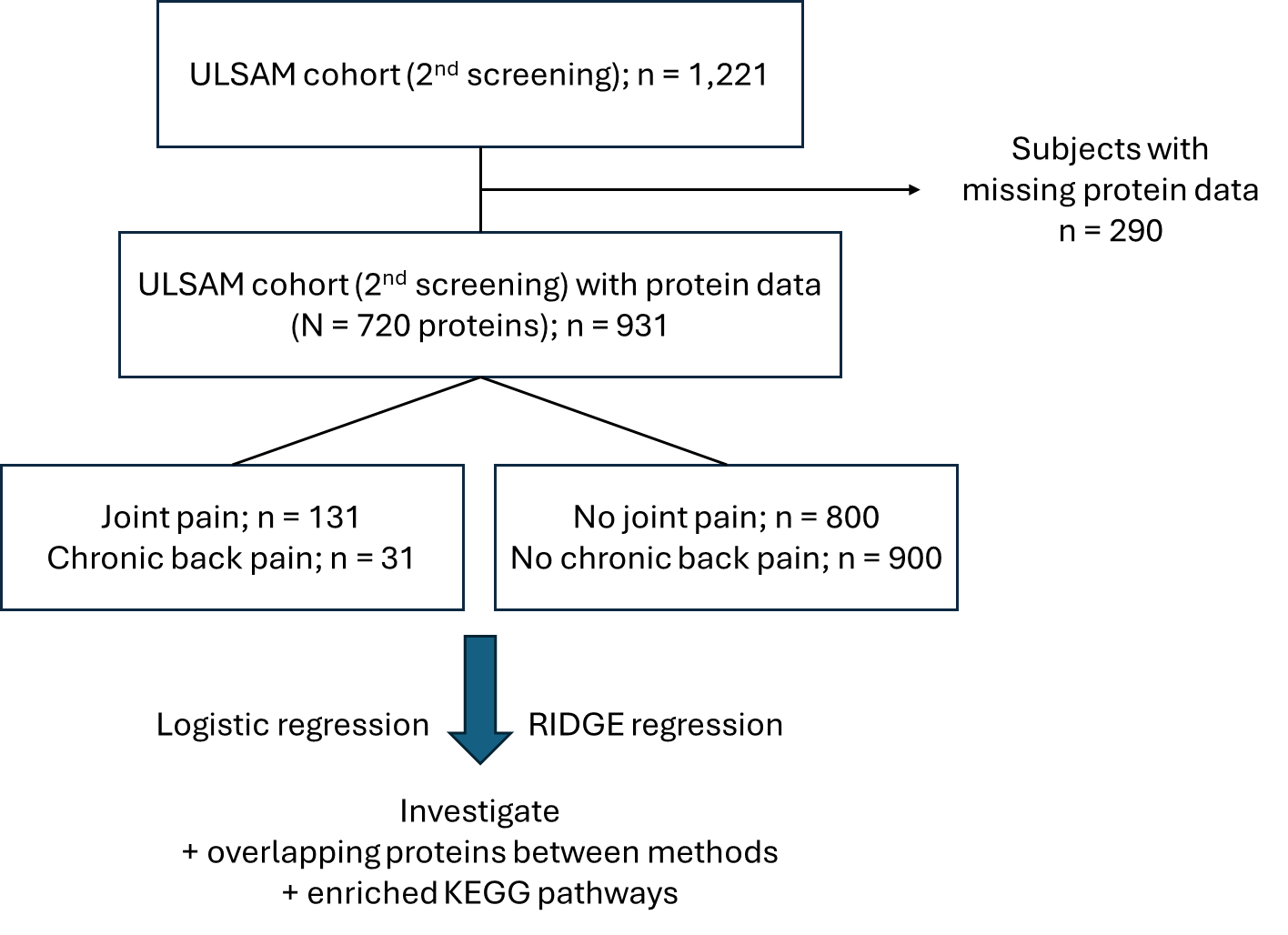


**Supplemental Figure 1.** Overview of number of subjects and data analysis.


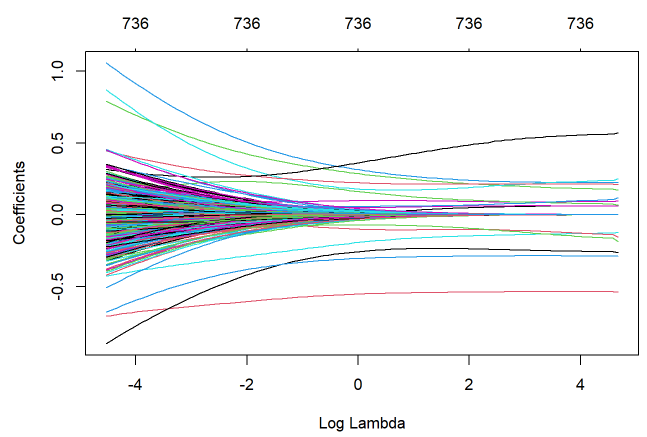

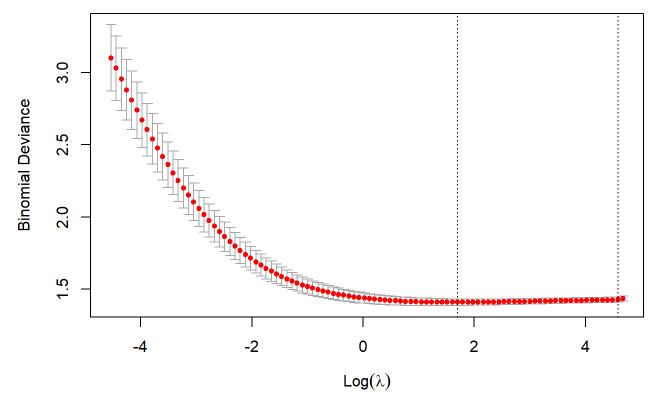


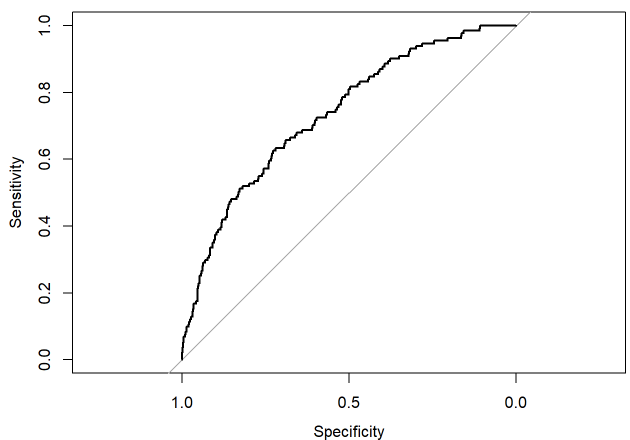

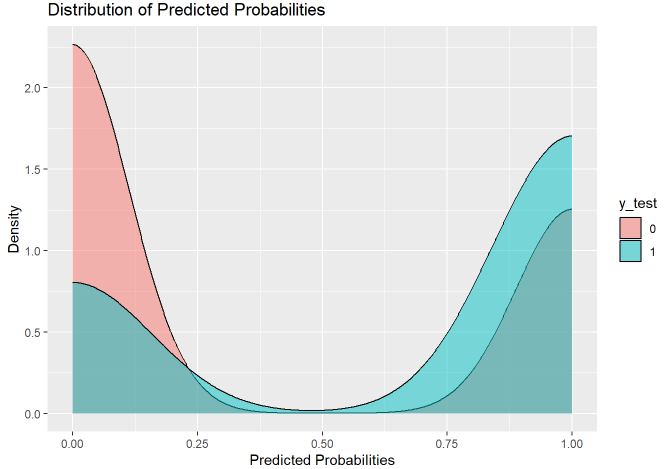


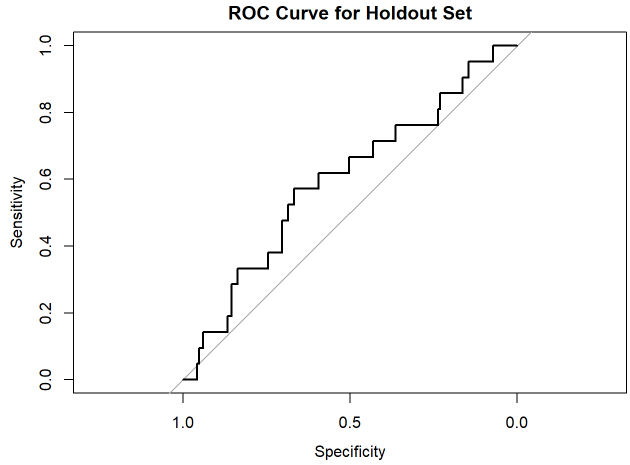

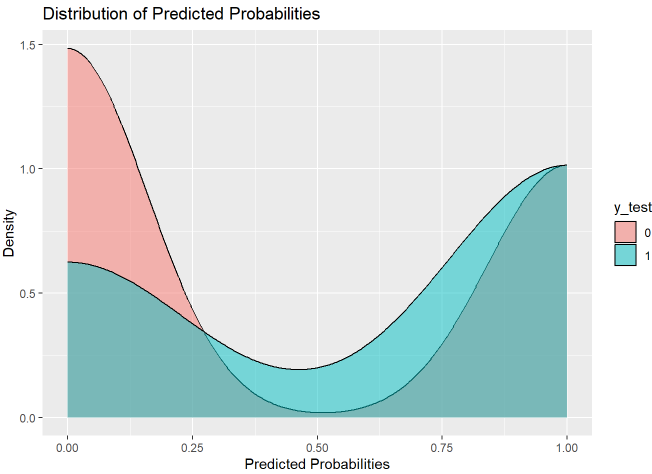


**Supplemental Figure 2**. RIDGE model visualization and performance metrics for joint pain and proteomics. **a)** Regularization plot, **b)** Cross-validation curve, **c)** ROC curve for training data (AUC = 0.73; prediction-recall curve AUC = 0.64), **d)** Distribution of predicted probabilities for training data, **e)** ROC curve for test data (AUC test = 0.60; prediction-recall curve AUC test = 0.67) and **f)** Distribution of predicted probabilities for test data.


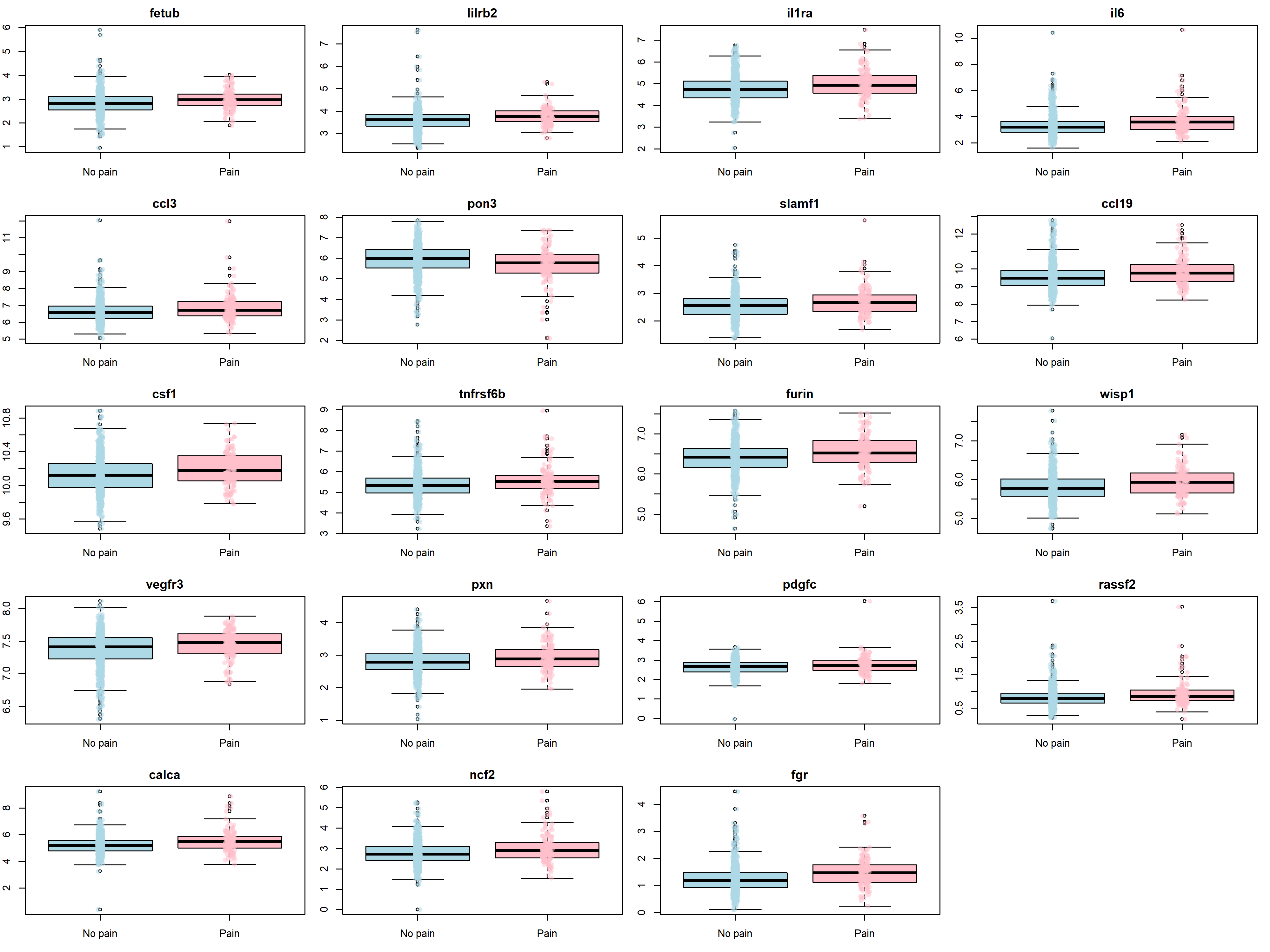


**Supplemental Figure 3**. Boxplots for 19 proteins associated with joint pain in both logistic regression (multiple testing adjusted significant) and in RIDGE regression (coefficient cut-off = |0.005|) with raw data points superimposed.

**
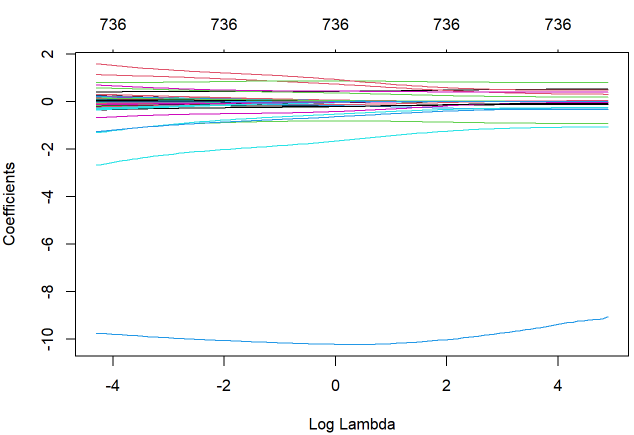

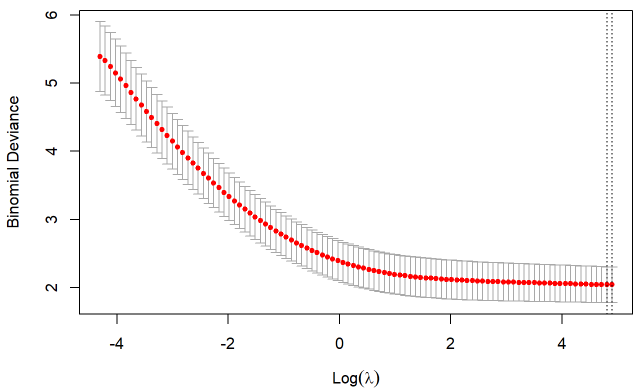
**

**
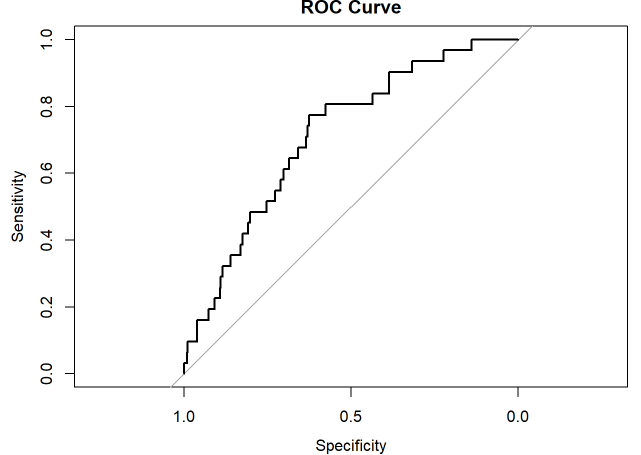
** **
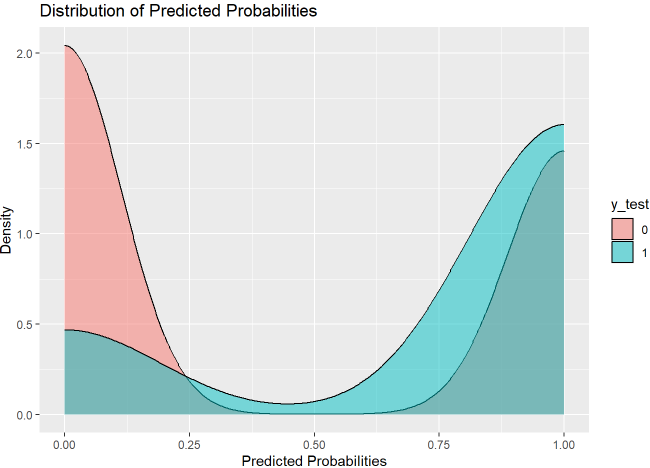
**

**
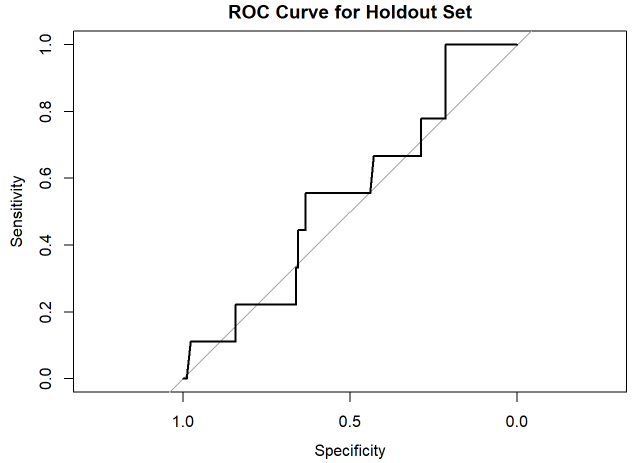
** **
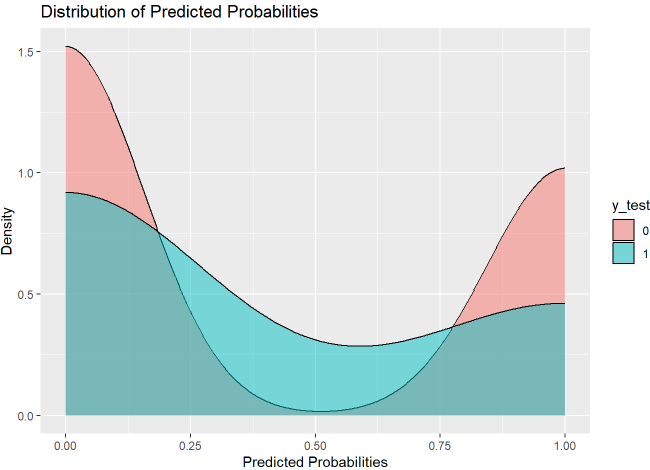
**

**Supplemental Figure 4**. RIDGE model visualization and performance metrics for chronic back pain and proteomics. **a)** Regularization plot, **b)** Cross-validation curve, **c)** ROC curve for training data (AUC = 0.71; prediction-recall curve AUC = 0.75), **d)** Distribution of predicted probabilities for training data, **e)** ROC curve for test data (AUC test = 0.55; prediction-recall curve AUC test = 0.72) and **f)** Distribution of predicted probabilities for test data.

**
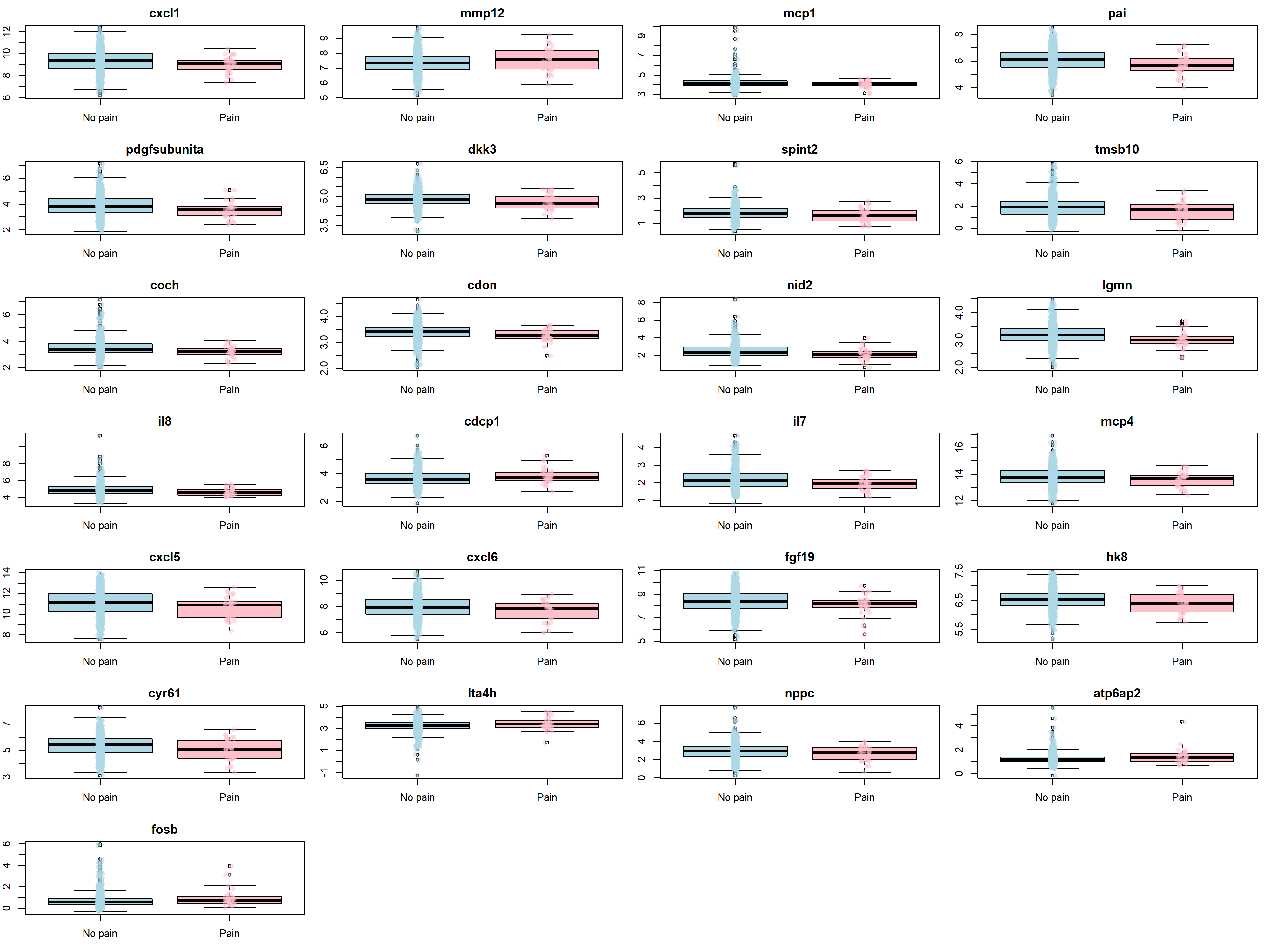
**

**Supplemental Figure 5**. Boxplots for 25 proteins associated with chronic back pain in both logistic regression (nominally significant) and in RIDGE regression (coefficient cut-off = |0.0005|) with raw data points superimposed.

**
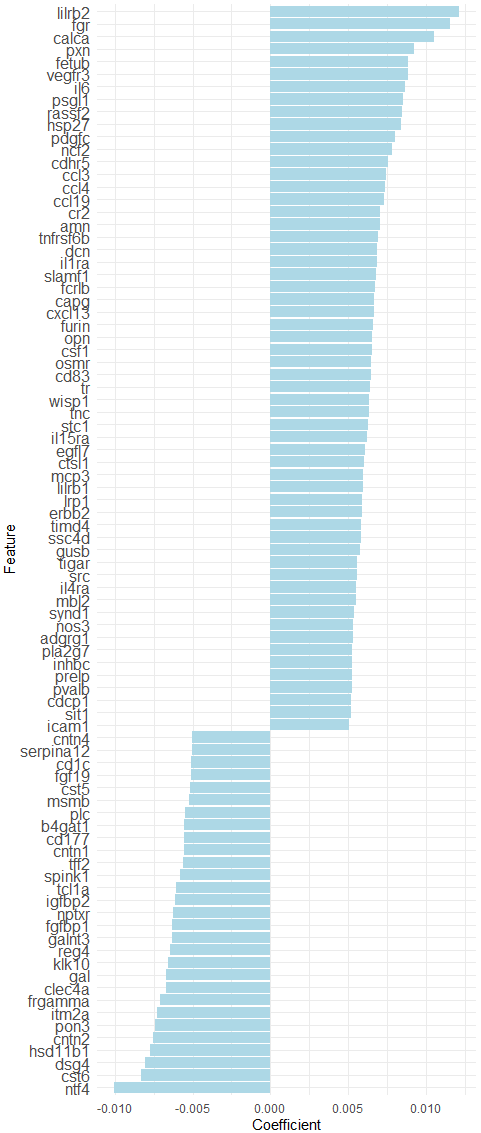
** **
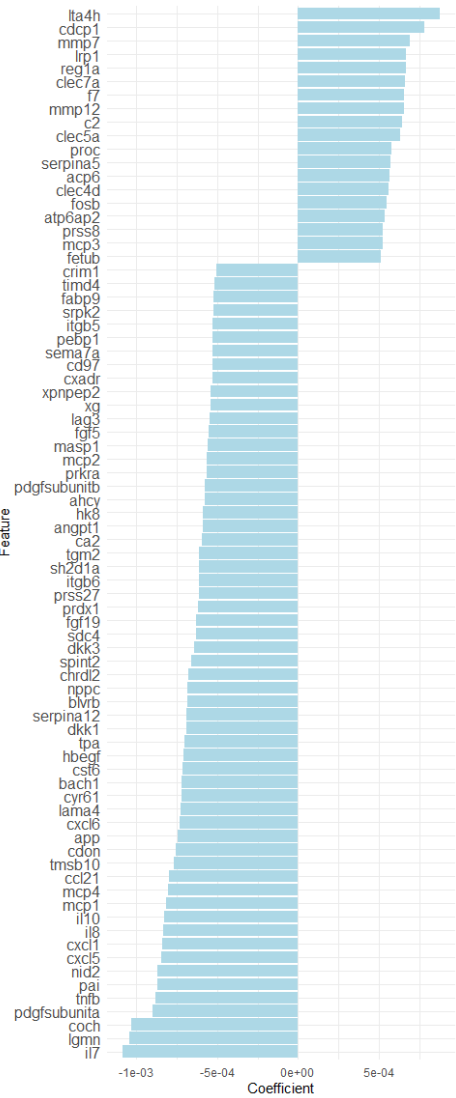
**

**Supplemental Figure 6.** Protein features selected in RIDGE regression with **a)** joint pain (coefficient cut-off = |0.005|) and **b)** chronic back pain (coefficient cut-off = |0.0005|).
